# Urinary excretion of gluten immunoreactive peptides as an indicator of gastrointestinal function after fasting and dietary provocation in healthy volunteers

**DOI:** 10.3389/fimmu.2024.1433304

**Published:** 2024-08-05

**Authors:** Raquel Rodríguez-Ramírez, María Auxiliadora Fernández Peralbo, Irati Mendía, Joshua C. D. Long, Carolina Sousa, Ángel Cebolla

**Affiliations:** ^1^ Research and Development Department, Biomedal S.L., Seville, Spain; ^2^ Inorganic Chemistry Department, Faculty of Science, University of Granada, Granada, Spain; ^3^ Department of Microbiology and Parasitology, Faculty of Pharmacy, University of Seville, Seville, Spain

**Keywords:** intestinal permeability, urine, gluten immunogenic peptides, lactulose, mannitol

## Abstract

**Introduction:**

Understanding intestinal permeability is paramount for elucidating gastrointestinal health and pathology. The size and nature of the molecule traversing the intestinal barrier offer crucial insights into various acute and chronic diseases, as well as the evolution of some conditions. This study aims to assess the urinary excretion kinetics of gluten immunogenic peptides (u-GIP), a unique class of dietary peptides detectable in urine, in volunteers under controlled dietary conditions. This evaluation should be compared to established probes like lactulose, a non-digestible disaccharide indicative of paracellular permeability, and mannitol, reflecting transcellular permeability.

**Methods:**

Fifteen participants underwent simultaneous ingestion of standardized doses of gluten (10 g), lactulose (10 g), and mannitol (1 g) under fasting conditions for at least 8 hours pre-ingestion and during 6 hours post-ingestion period. Urine samples were collected over specified time intervals. Excretion patterns were analyzed, and correlations between the lactulose-to-mannitol ratio (LMR) and u-GIP parameters were assessed.

**Results:**

The majority of u-GIP were detected within the first 12 hours post-ingestion. Analysis of the variability in cumulative excretion across two sample collection ranges demonstrated that lactulose and u-GIP exhibited similar onset and excretion dynamics, although GIP reached its maximum peak earlier than either lactulose or mannitol. Additionally, a moderate correlation was observed between the LMR and u-GIP parameters within the longest urine collection interval, indicating potential shared characteristics among permeability pathways. These findings suggest that extending urine collection beyond 6 hours may enhance data reliability.

**Discussion:**

This study sheds light on the temporal dynamics of u-GIP in comparison to lactulose and mannitol, established probes for assessing intestinal permeability. The resemblance between u-GIP and lactulose excretion patterns aligns with the anticipated paracellular permeability pathway. The capacity to detect antigenic food protein fragments in urine opens novel avenues for studying protein metabolism and monitoring pathologies related to the digestive and intestinal systems.

## Introduction

The integrity of the epithelial barrier within the intestine is a crucial determinant of the pathogenesis of various gastrointestinal diseases. Assessing intestinal permeability is essential for elucidating the origin of symptom in undiagnosed patients, monitoring gastrointestinal disorders, and investigating the role of the intestine in multifarious diseases (1). Intestinal permeability enables a balanced exchange of fluids and solutes between the intestinal lumen and bloodstream, constituting a key characteristic of the protective barrier (2). Regulation of molecular passage occurs through transcellular absorption and paracellular absorption, mediated by tight junctions between intestinal epithelial cells (3). Gut barrier dysfunction has been associated not only with chronic gastrointestinal conditions like inflammatory bowel disease or irritable bowel syndrome, but also with metabolic disorders, alcoholic liver disease, chronic arthritis, and neuropsychiatric disorders (4). Generally, transport mechanisms in the intestinal mucosa allow the passage of amino acids, dipeptides, and tripeptides and limited quantities of larger peptides via transcytosis after binding to receptors on the intestinal membrane. These peptides pose a high risk of acting as antigens and consequently contributing to the development of food allergies and intolerance in the event of intestinal barrier dysfunction (5).

The investigation into gluten, a complex mixture of proteins called prolamins, present in wheat, barley, rye, oats, and their derivatives has been driven by its association with celiac disease. The incomplete digestion of gluten results in proline-rich, digestion-resistant immunogenic peptides. These peptides, hereafter referred to as GIP (gluten immunogenic peptides), can be detected in feces (6) and urine (7). The presence of GIP in the urine of healthy individuals after gluten consumption suggests they can cross the intestinal epithelium into the bloodstream, undergo filtration by the kidney, and ultimately be excreted in the urine (5). Given the size of detectable GIP, they are likely to traverse the paracellular pathway, which is compromised in individuals with increased intestinal permeability to large molecules. This renders them as a unique model for studying the absorption of biologically active macromolecules, that can be monitored at various metabolic stages using diverse methodologies (8).

The determination of intestinal permeability has long been a topic of debate in scientific circles. Established methodologies involve the oral administration of tracer molecules, such as labeled chromium-ethylenediaminetetraacetic acid (51Cr-labeled EDTA), or non-digestible sugars, such as lactulose (lac) and mannitol (man), followed by the analysis of urinary excretion. Although these tests have been utilized for decades, they suffer from drawbacks including time-intensive procedures, lack of standardization, inability for retrospective analysis, and limited validity due to uncertainties surrounding normal reference standards (9).

The lactulose/mannitol test, designed to discern paracellular and transcellular absorption dynamics, involves orally administering a solution containing lactulose, a disaccharide which cannot cross an intact epithelia, and mannitol, a monosaccharide capable of transcellular transport (10). After ingestion, the urinary excretion of lactulose and mannitol is quantified over a specified duration, with the resulting lactulose-to-mannitol ratio (LMR) serving as a surrogate marker of intestinal permeability (11). In conditions where intestinal permeability is increased, as is commonly observed in various gastrointestinal disorders, a higher LMR indicates increased transfer of substances across the intestinal suggesting compromised epithelial barrier integrity (12). Despite its widespread utilization, the lactulose/mannitol test has limitations. It measures the permeability of small sugar molecules that lack immunogenic activity, thus not allowing analysis of the ability of antigenic macromolecules to pass the epithelial barrier, which can cause and exacerbate underlying inflammatory conditions and autoimmune diseases. Additionally, lactulose has a low molecular weight, and the transfer of this substance through the intestinal barrier does not reflect the transfer of dietary proteins and the overall immune response (lactulose, 382 Da; mannitol, 182 Da; gliadin peptides, ~ 2000-4000 Da) (13). Moreover, establishing normative LMR thresholds for healthy and diseased states remains a subject of ongoing investigation, highlighting the need for further refinement and standardization within the realm of intestinal permeability assessment methodologies. Furthermore, researchers have explored the potential of various endogenous proteins such as lipopolysaccharide-binding protein or zonulin as biomarkers for intestinal permeability. However, consensus regarding their clinical utility has yet to be reached (4).

Multiple observations indicate that in conditions in which intestinal function/health is compromised, GIP are able to cross the intestinal epithelium more readily, likely due to increased permeability. Firstly, it has been observed that in celiac patients consuming equivalent gluten challenges, there was a variation in the amount of urinary GIP detected and those patients with the highest level of uGIP showed a greater degree of villus atrophy progression (unpublished results). Additionally, utilizing a specific peptidomics workflow using LC-MS, urine samples from celiac patients at diagnosis have shown up to four times higher number of gluten peptides compared to those from healthy volunteers (14). These preliminary findings suggest that quantifying GIP excreted in urine could serve as an indicator of intestinal permeability to immunogenic macromolecules. In this study, we investigated the kinetic and dynamic patterns of u-GIP excretion after simultaneous consumption of gluten, lactulose, and mannitol to explore pathways of epithelial barrier translocation and preliminarily assess its potential as a standard food antigen probe in intestinal permeability analysis.

## Materials and methods

### Study population

Fifteen healthy volunteers were included using the criteria: (1) age >18 years; (2) absence of diagnoses for CD, non-celiac gluten sensitivity, food allergies, food intolerances, or other gastrointestinal diseases; (3) willingness to adhere to a strict diet regimen; and (4) capability to collect daily urine samples. Exclusion criteria included: (1) presence of concurrent pathologies. Participants failing to collect samples correctly on at least 70% of occasions were not excluded during participant recruitment but were excluded during the analysis phase. The study was conducted according to the guidelines of the Declaration of Helsinki. All participants provided written informed consent, and the study received approval from the local ethics committee (n. 1308-N-23).

### Study design

**Figure 1:**
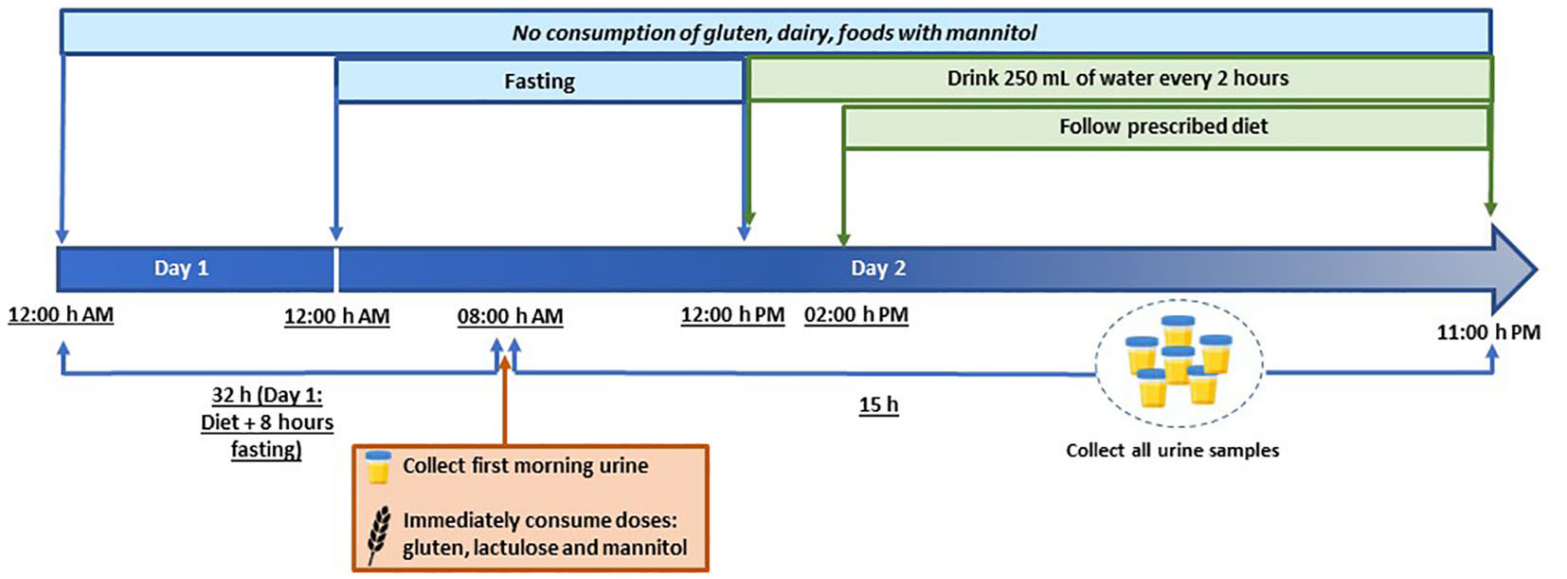
Study timeline showing the periods of fasting, gluten/lactulose/mannitol consumption, liquid consumption and sample collection.

The study comprised two stages: a washout period and an intake/collection period (**Figure 1**). The washout period lasted for 32 hours (including a final 8 hours fasting period) during which volunteers adhered to a gluten-free diet and abstained from dairy and foods high in sorbitol and/or mannitol. Before ingestion, volunteers provide a urine sample to confirm the absence of target compounds. Gluten (10 g), lactulose (10 g), and mannitol (1 g) were ingested after a period of eight hours fasting. Participants fasted for the initial 4 hours post-ingestion, after which they commenced scheduled liquid intake (250 mL every 2 hours). At the 6-hours mark, participants began the prescribed diet. The study concluded 15 hours post-ingestion of the compounds.

Throughout the study, diet adherence and fluid intake were assessed using a food recall questionnaire, and participants recorded daily food consumption details. A schematic of the study timeline is illustrated in **Figure 1**.

### Compounds administration

The compounds comprised 10 g of gluten powder (Aurora Intelligent Nutrition, Sevilla, Spain), an oral solution sachet of lactulose (Duphalac™, Abbott Laboratories, S.A., Madrid, Spain), and 1 g of mannitol (Acofarma, Madrid, Spain). For mannitol intake, 50 mL of water was added to the tube and ingested after shaking.

To administer the gluten intake, a portion of the content was suspended in 125 mL of water, post-ingestion, an additional 125 mL of water was added to ensure complete suspension of the remaining powder.

### Urine collection

Comprehensive instructions were provided to all participants at the study’s commencement. Subjects were equipped with all necessary materials for urine collection, including plastic screw-capped containers, labels, cool bags, isothermal boxes, and cool packs. Participants were instructed to collect the entire urine sample from each micturition, noting the date and time of collection. All urine samples were preserved in isothermal boxes with cool packs at 4–8°C and deposited within 48 hours of collection. Samples were then frozen at -20°C until processing. GIP concentration in urine remained stable throughout the freeze-thaw process.

### Urine analysis

The volume of each urine sample was recorded, and when multiple containers were required for the same urination, they were mixed and homogenized. Additionally, some mixed samples were analyzed at intervals of 0–6-hours and 2–15-hours. To create these mixtures, 10% of the volume of each container was utilized. To prevent bacterial growth, 100 µL of 1% chlorhexidine diacetate was added. Aliquots of 1 mL were done and stored at -20°C until analysis.

#### u-GIP analysis

Qualitative analysis of GIP in urine was conducted using a lateral flow immunoassay (LFIA) with iVYCHECK GIP Urine (Biomedal S.L., Seville, Spain), following the manufacturer’s guidelines. Thawed urine samples were homogenized and mixed with a conditioning solution. Subsequently, 100 µL of the mixture was added to the immunochromatographic cassette, and visual interpretation of results occurred after 30 minutes. A positive outcome was determined if the test line exhibited a red color accompanied by a green color on the control line. A negative result was confirmed when only the control line displayed a green color. The Limit of Detection (LoD) determined by visual inspection was 2.50 ng GIP/mL urine. GIP concentration in urine was also assessed on the immunochromatographic strips using the iVYCHECK Reader (Biomedal S.L., Seville, Spain). Reader calibration was performed before urine analysis using the α-gliadin 33-mer peptide as a standard. The measuring range established for this method was 3.12–25 ng GIP/mL urine. Results were expressed as ng GIP per mL of urine. Each sample underwent duplicate runs, and at least two aliquots of each sample were tested.

#### Lactulose/Mannitol analysis

The determination of lactulose and mannitol analytes was conducted using a method developed and validated by LC-MS/MS, with a linear range from of 10–1200 mg/L. The LC-MS/MS system comprised an HPLC coupled a triple quadrupole mass spectrometer (QSight 220, Perkin Elmer™, Waltham, Massachusetts, USA), equipped with an electrospray ion source.

HPLC separation was performed using a 100 x 2.1 mm, Hypercarb™ column (Thermo Scientific, Waltham, Massachusetts, USA), operating at a flow rate of 0.25 mL/min. Elution was carried out with a 25-minute linear gradient from 1 to 4% acetonitrile in water containing 0.1% formic acid, with the oven temperature set at 11°C. The injection volume was 5 *µ*L, and the total analysis time was 27 minutes.

Multiple Reaction Monitoring (MRM) was employed, with parent and fragments ions monitored at Q1 and Q3, respectively. Optimization of parent and daughter ions, along with collision voltages, was conducted through experiments where pure standards were infused into the mass spectrometer in the mobile phase. The ESI source operated in the negative mode with the following mass parameters: Drying Gas, 120 (arbitrary units); Hot-Surface Induced Desolvation (HSID) Temperature, 250°C; Nebulizer gas, 350 (arbitrary units); Electrospray voltage, -5500 V; and Source Temperature, 300°C.

Aliquots were thawed and agitated for one minute using a vortex mixer. Subsequently, they were centrifuged for 5 minutes at 5000 g to remove sediments. Fifty microliters of the supernatant were collected and brought to a final volume of 1 mL with water. After shaking, the same dilution was repeated, with an additional 50 µL of internal standard (2 mg/mL melezitose) added. The final dilution was 1:400 (v/v).

### Statistical analysis

Quantitative variables results are presented using both the mean (SD) and median (IQR or range), while categorical variables are expressed as absolute (N) and cumulative (%) frequencies. The LMR values were then multiplied by 100. Interquartile tests were performed using RStudio (Version 2022.02.3 + 492, RStudio, Inc., Boston, MA, USA), and correlation tests were conducted using Microsoft Excel (Version 2401, Microsoft Corporation, Redmond, WA, USA).

## Results

### Participants and samples

Fifteen individuals, comprising 12 (80%) females and three (20%) males, completed the study. The median age of participants was 35 years (IQR 29–41). None of the participants had been diagnosed with any relevant diseases. Based on exhaustive food recall data, including specific brands and detailed consumption information, all participants adherent to the prescribed diet and consumed the provided compounds.

### Urine analysis

Throughout the study, 107 urine samples were collected from all participants. To ensure data integrity, rigorous exclusion criteria were applied, in fact, five samples were excluded due to their duration exceeding the predefined study window.

It is essential to emphasize the meticulous attention paid to the mixing process, particularly regarding urine samples collected at 2–15-hours intervals. While most samples adhered strictly to the study protocol, exceptions were made for two volunteers urines samples during the 2–15-hours interval mixing due to their temporal deviation beyond the stipulated range. This decision was justified by the time gap between the last urination before the temporal limitation and the subsequent excretion, warranting their inclusion for measurement purposes. Similar exceptions were noted during the 0–6-hour pooling process, totaling three instances.

Considering the aforementioned exceptions the statistical analyses provided valuable insights into the mixing procedures. For the 0–6-hours interval, the median number of urine samples utilized for mixing was 2.00 (IQR 2-3), with a mean pooled six-hourly urine volume of 348 mL ± 176 mL. In contrast, during the 2–15-hours interval, there was a median of six urine samples (IQR 4-6) employed for mixing, resulting in an average total volume of 983 mL ± 331 mL.

Excluding baseline urination, the average number of urinations per participant was determined to be 6 (IQR 5-7). Importantly, baseline urinations showed non-detectable levels of u-GIP and lactulose, indicating the successful adherence to the pre-study dietary requirements. However, an average of 25 ppm ± 17 ppm of mannitol was detected in these samples, suggesting the challenges associated with adhering to a mannitol-free diet, despite recommendations.

### Excretion kinetics

**Figure 2:**
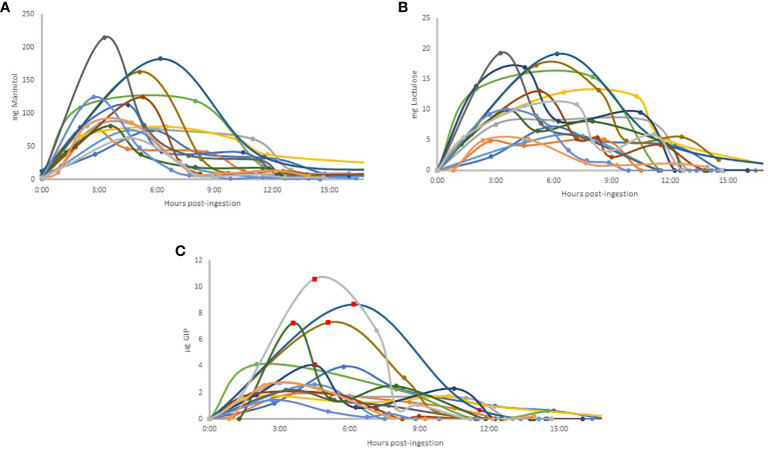
Individual excretion patterns in urine: Mannitol **(A)**; Lactulose **(B)**; GIP **(C)**. Each volunteer is represented by a color that is the same for all three compounds studied. In Figure C, each value extrapolated above the upper limit for accurate quantification of GIP is represented by a red square on the graph, based on the standard curve, for illustrative purposes.Abbreviations: GIP, Gluten Immunogenic Peptides.

The excretion kinetics of u-GIP were investigated following ingestion, revealing insightful patterns among participants. On average, the initial urine sample with detectable levels containing GIP was excreted 2 hours and 53 minutes after ingestion, with most volunteers producing this sample as their first urine output following ingestion. The average peak excretion time was 4 hours and 08 minutes. Notably, exceptions were observed in two volunteers, whose first urine occurred approximately one hour after ingestion. These samples tested negative for GIP, possibly suggesting incomplete bladder emptying before ingestion. The detectable excretion of GIP extended to 9 hours and 43 minutes post-ingestion, with the first undetectable urine excreted, on average, at 12 hours and 55 minutes post-ingestion. Intriguingly, a double excretion peak was identified in 40% of volunteers, suggesting potential variability in u-GIP excretion dynamics. Above the upper limit of quantification (values represented by red squares in **Figure 2**), approximate concentrations were used based on extrapolation from the standard curve.

Analysis of lactulose excretion kinetics (**Figure 2**) revealed distinct temporal dynamics among participants. The first positive lactulose urine sample was typically excreted approximately 2 hours and 44 minutes post-ingestion, closely aligning with the timing of u-GIP excretion. Similar to u-GIP excretion, the first urine after ingestion coincided with the first urine with detectable levels, except for one volunteer, whose first urine after ingestion occurred just one hour later and showed a negative result for lactulose, possibly indicating incomplete bladder emptying during lactulose ingestion. The average for the peak excretion time was 5 hours and 38 minutes. Lactulose excretion persisted for an average of 10 hours and 21 minutes post-ingestion, with the first urine with undetectable levels appearing, on average at 13 hours and 23 minutes post-ingestion. Intriguingly, lactulose did not reach negativity during the study for two volunteers, suggesting distal absorption of the molecule. Moreover, a double excretion peak was evident in 46.67% of volunteers, indicating potential variability in lactulose absorption/excretion patterns.

As mentioned previously, all baseline urine samples exhibited quantifiable values for mannitol (**Figure 2**). The average peak excretion time was 4 hours and 52 minutes. Notably, a double excretion peak was identified in 40% of volunteers, with two volunteers exhibiting double excretion peaks for all three compounds studied. None of the volunteers reached negativity during the study (**Figure 2**).

The comparison of the time after ingestion at which the peak excretion occurred for each compound and each volunteer, as well as the corresponding urine number, was analyzed (Table I). For all three compounds, the peak excretion (on average for all participants) occurred in the third urine sample. In 50% of volunteers, the time and, consequently, the urine matched for all three compounds. In 81% of volunteers, the excretion peaks for lactulose and mannitol coincided in the same urine sample.

### Analysis of the variability in cumulative excretion within two ranges of sample collection

The excretion percentages for lactulose and mannitol were calculated based on both the ingested and total determined excreted quantities. Subsequently, the percentages of both compounds excreted in the 0–6-hours and 2–15-hours interval mixtures were determined. The excretion percentage in the 2–15-hours interval mixture was considered as 100%, and the proportion of this mix excreted within the 0–6-hours window was then calculated. For lactulose, the mean excretion rate was found to be 56.54% ± 16.53%, while for mannitol, the mean excretion rate was observed to be 72.54% ± 16%.

Regarding the excretion of u-GIP, due to the unavailability of precise ingestion data and the uncertainty surrounding the exact percentage of GIP in the 10 g of gluten consumed, the u-GIP quantity is utilized for calculations by using α-gliadin 33-mer (a principal contributor to gluten immunotoxicity) (15) as a standard. Employing the same methodology as with the aforementioned compounds, a mean excretion rate of 55.97% ± 20.66% is derived for the 0–6-hours interval mixture, with 100% representing the excretion in the 2-15-hours interval mixture.

The Lactulose-to-Mannitol ratio (LMR) was calculated for each participant in the 0–6-hours and 2–15-hours interval mixtures, yielding means of 1.04 ± 0.36 and 1.43 ± 0.53, respectively. Individual coefficients of variation between the ratios of each time interval were calculated for each participant, resulting in a mean of 18.81% ± 14.33%. The correlation graph between the LMR of the two intervals demonstrated a moderate correlation with an R^2^ value of 0.5977.

### Outliers’ analysis

**Figure 3:**
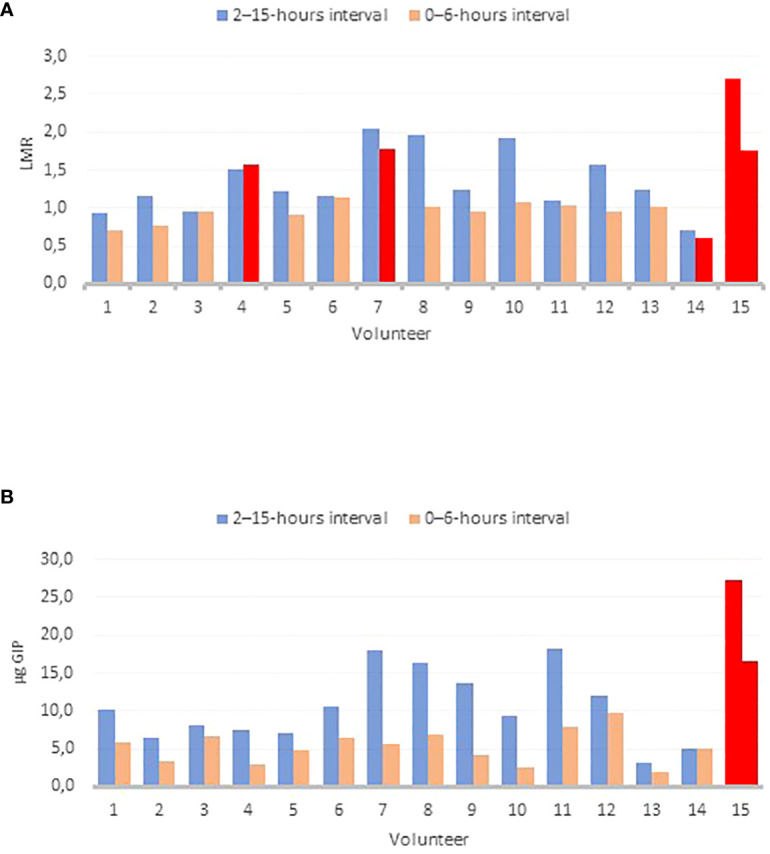
Outlier’ plots for different parameters: LMR 0–6 hours and 2–15-hours intervals **(A)**; GIP 0–6-hours and 2–15-hours interval **(B)** The Interquartile Range (IQR) method was utilized for outlier detection. Outlier’ results are shown in red. Abbreviations: LMR, lactulose-to-mannitol ratio; GIP, Gluten Immunogenic Peptides.

A study on outliers was conducted with the volunteers to determine if any participant demonstrated outlier values in the assessed parameters (LMR and u-GIP) at different time intervals (**Figure 3**). Out of the four parameters analyzed, volunteer 15 consistently exhibited outlier values across all parameters, while three other volunteers showed outlier values specifically in the LMR parameter within the 0–6-hours interval. Outlier calculations were performed using the Interquartile Range (IQR) method.

### Analysis of the correlation between the different parameters

Considering that LMR is the most commonly used methodology for assessing intestinal permeability, it served as the reference control for analyzing u-GIP metabolism, particularly the absorption/excretion kinetics. Scatterplots were generated to compare the data from all volunteers for the different compounds. In addition to comparing the quantities of LMR and u-GIP at the two-time intervals, the correlation was also studied using only the percentage of lactulose excreted. A moderate correlation was observed between the LMR and u-GIP quantity in the 2–15-hours interval (R^2^ = 0.5225), inclusive of outliers. When the same representation but disregarding outlier results is done, only lactulose vs u-GIP comparison in the 2–15-hours interval shows a certain correlation (R^2 =^ 0.4159). There is no correlation observed in the 0–6-hours interval.

## Discussion

In this study, we evaluated the excretion dynamics of lactulose and mannitol, widely used probes for assessing intestinal permeability, in urine samples collected from healthy volunteers who consumed controlled doses under regulated dietary conditions. Furthermore, simultaneous consumption of gluten in addition to lactulose and mannitol was conducted to explore the pathways of epithelial barrier translocation of gluten peptides and evaluate their potential as a biomarker in intestinal permeability analysis.

GIP represent a heterogeneous mixture of peptides of varying sizes and degrees of hydrolysis. The assay used can detect both small peptides and intact gliadin protein. Given the heterogeneous mix of gluten digestion products, the relative quantification of GIP concentration is provided in reference to a standard curve of the immunogenic 33-mer GIP peptide. It is important to note that the detected signal does not exclusively indicate the presence of this specific peptide.

One notable finding was the comparative ease of adhering to a short-term gluten-free diet over abstaining from mannitol-containing foods, supported by the analysis of baseline urine samples from all participants. The straightforward identification of gluten sources contrasted with the complexity of identifying and avoiding various mannitol-rich foods (16). Despite instructions to avoid mannitol consumption during the washout period, all participants exhibited detectable concentrations of mannitol in their urine samples before ingesting the pure compound. Taking into account the average mannitol value found in baseline urines (25 ppm ± 17 ppm) and the average values for all volunteers in the different intervals (638 ppm ± 366 ppm for the 0–6-hours interval and 291 ppm ± 42 ppm for the 2–15-hours interval), it is concluded that the contribution of baseline urine is insignificant.”

Our results revealed a median duration of u-GIP excretion of approximately 10 hours, with the first urine with undetectable levels sample collected, on average, around 13 hours post-ingestion. These findings align with those of Coto et al. (7), who reported u-GIP detection within 1–15 hours after ingesting 2 grams of gluten in a homogeneous breakfast among their study participants. Notably, our study observed a mean peak in u-GIP excretion approximately 4 hours post-ingestion, contrasting with the findings of Coto et al. (7) of a peak between 6 and 9 hours. This discrepancy may stem from differences in the amount and the form of gluten ingested. Unlike the previous study, which did not incorporate a fasting period after gluten ingestion, our participants consumed gluten on an empty stomach in dispersed powder form. In contrast, Coto et al. (7) demonstrated that gluten was administered in capsules with breakfast, requiring metabolism alongside the meal. These results underscore the efficacy of administering gluten in suspension after a fasting period, enhancing metabolic efficiency.

In comparison, Moreno et al. (17) reported a narrower detection window for u-GIP, with detectable levels observed between 3- and 15-hours post-ingestion. Furthermore, they noted an extended time for u-GIP disappearance after a normal gluten-containing diet, ranging from 16 to 34 hours, compared to our study. These differences highlight the influence of gluten dosage and dietary context on u-GIP excretion dynamics and/or the higher sensitivity owing to the previous solid-phase extraction protocol of the samples.

**Figure 4:**
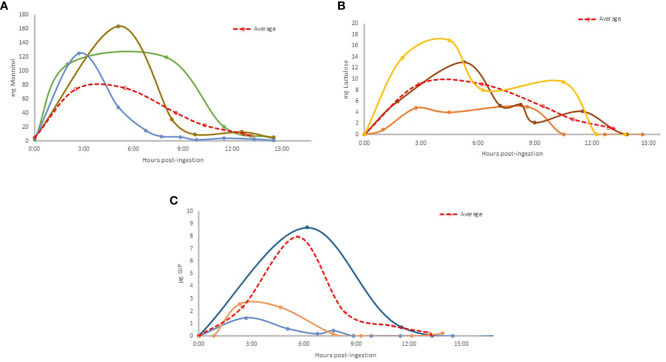
Excretion patterns of selected volunteers where: Mannitol excreted **(A)** (Light blue: Vol. 13; Brown: Vol.10; Green: Vol. 6); Lactulose excreted **(B)** (Brown: Vol. 8; Orange: Vol. 2; Yellow: Vol. 7); u-GIP excreted **(C)** (Light blue: Vol. 13; Orange: Vol. 14; Dark blue: Vol. 11). The average results are shown in a dashed red line.

Our study provides novel insights into the temporal patterns of u-GIP excretion following gluten ingestion under fasting conditions. The observed variations underscore the complex interplay between gluten exposure and GIP metabolism, with potential implications for gastrointestinal physiology and metabolic health. When the excretion patterns of specific volunteers were analyzed (**Figure 4**), significant heterogeneity was observed. The plots illustrate the variance between individuals with different excretion rates, including instances of double-peaked excretion profiles.

A comparison of the excretion dynamics between lactulose/mannitol and u-GIP revealed remarkable similarities, particularly in the temporal patterns observed. Both lactulose and u-GIP exhibited comparable times to the first urine with quantifiable levels sample post-ingestion, with lactulose detected at 2 hours and 44 minutes and u-GIP at 2 hours and 53 minutes, suggesting a similar excretion dynamic.

Similarly, the duration of excretion, as indicated by the time to the first urine with undetectable levels sample post-ingestion, remained consistent for lactulose (13 hours and 23 minutes) and u-GIP (12 hours and 55 minutes), emphasizing their parallel kinetics. As mentioned previously, all baseline urine samples tested positive for mannitol, indicating pre-existing exposure, and none reached undetectable levels during the study period. Notably, the peak excretion times differed slightly, with lactulose and mannitol peaking at approximately 5 hours, and u-GIP peaking at 4 hours and 8 minutes. These findings underscore the feasibility of the GIP-based test, as a reliable marker of intestinal permeability, with the advantage of utilizing a real food antigen that may be implicated in immunological disorders and without transgressions by the participants.

Analyzing the findings presented in **Table 1**, it can be concluded that while excretion patterns varied among individual volunteers—some exhibiting early excretion peaks at 2 hours post-ingestion and others at 8 hours post-ingestion—it is notable that the third urine sample corresponded to the time of maximum concentration for all three compounds. This finding could inform the development of more accurate protocols and interpretation of future studies related to the excretion kinetics of these compounds.

**Table 1:** Characteristics of peak excretion time for each compound and volunteer.

	Component	Peak time (h)	Urine number		Component	Peak time (h)	Urine number
**Volunteer 1**	**Mannitol**	5:45	3	**Volunteer 9**	**Mannitol**	3:15	2
	**Lactulose**	5:45	3		**Lactulose**	3:15	2
	**GIP**	5:45	3		**GIP**	3:15	2
**Volunteer 2**	**Mannitol**	2:43	3	**Volunteer 10**	**Mannitol**	5:05	2
	**Lactulose**	8:35	5		**Lactulose**	5:05	2
	**GIP**	4:28	2		**GIP**	5:05	2
**Volunteer 3**	**Mannitol**	6:00	3	**Volunteer 11**	**Mannitol**	6:10	2
	**Lactulose**	6:00	3		**Lactulose**	6:10	2
	**GIP**	3:00	2		**GIP**	6:10	2
**Volunteer 4**	**Mannitol**	6:30	3	**Volunteer 12**	**Mannitol**	3:35	3
	**Lactulose**	6:30	3		**Lactulose**	3:35	3
	**GIP**	2:00	2		**GIP**	3:35	3
**Volunteer 5**	**Mannitol**	4:30	2	**Volunteer 13**	**Mannitol**	2:41	2
	**Lactulose**	7:30	3		**Lactulose**	2:41	2
	**GIP**	7:30	3		**GIP**	2:41	2
**Volunteer 6**	**Mannitol**	8:00	3	**Volunteer 14**	**Mannitol**	4:40	4
	**Lactulose**	8:00	3		**Lactulose**	4:40	4
	**GIP**	2:00	2		**GIP**	2:20	3
**Volunteer 7**	**Mannitol**	4:30	3	**Volunteer 15**	**Mannitol**	4:30	3
	**Lactulose**	4:30	3		**Lactulose**	7:10	4
	**GIP**	4:30	3		**GIP**	4:30	3
**Volunteer 8**	**Mannitol**	5:15	3	**Media**	**Mannitol**	4:52	2,7
	**Lactulose**	5:15	3		**Lactulose**	5:38	3,0
	**GIP**	5:15	3		**GIP**	4:08	2,6

GIP, Gluten Immunogenic Peptides.

The presence of volunteers with multiple excretion peaks aligns with existing literature, where the temporal curves for the percentage excretion of mannitol and lactulose exhibited a bimodal pattern with early and later peak (11). In the previous study, the initial peak in mannitol excretion occurred between one- and two-hours post-dosage. However, in our data, the first peak for mannitol was observed at around 4.5 hours, which does not coincide with their findings. We did observe a similarity with the second peak for lactulose at 4 hours, which corresponds to the first peak in our study. Since the previous study only collected data up to 6 hours, it is unknown whether a later peak would have appeared, as we found in our results at 10:36 for lactulose and 12:10 for mannitol. Additionally, we detected one case with up to three excretion peaks, indicating further interindividual variations in the absorption and excretion of these compounds. These findings underscore the intricate interplay between excretion dynamics and urine characteristics, highlighting the complexity of excretion dynamics and the need to consider individual variability in future studies on intestinal permeability.

The urinary levels of GIP were notably within the µg range, while those of lactulose were in the mg range, despite similar intake quantities. This discrepancy suggests that gluten peptides demonstrate lower permeability than lactulose and/or undergo significantly greater hydrolysis, rendering them more challenging to detect. However, the immunogenicity of gluten peptides holds greater biological relevance than saccharide molecules when analyzing potential systemic inflammatory responses.

Two different collection intervals were tested. Firstly, 2–15-hours interval was chosen because it is documented as the period during which the majority of GIP are excreted (7). Thus, this collection period should capture the vast majority of GIP excreted following the 10g gluten. However, it was determined, that collecting urine for 15 hours would be logistically challenging for patients necessitating the testing of a shorter collection time frame. Given that multiple studies collect urine for 6 hours to measure lactulose/mannitol (18), the 0–6-hour period was also selected.

Different time intervals for urine collection offer varied interpretations of the lactulose and mannitol test, depending on excretion time and gastrointestinal tract location. According to Odenwald et al. (19), the period from 0 to 2 hours after ingestion reflects small intestinal permeability. Between 2 to 8 hours after ingestion, markers are distributed in both the small and colonic intestines, indicating their combined presence in these regions. However, urine collected between 8 and 24 hours provides a more accurate reflection of colonic permeability. It is crucial to recognize that during this later period, colonic microbiota may degrade lactulose and mannitol, limiting their effectiveness in evaluating colonic permeability (20).

Our results, comparing the cumulative excretion within the 0–6-hours range against the 2–15-hours interval, revealed that lactulose and u-GIP did not reach the 60% excretion mark, whereas mannitol exceeded the 70% threshold. Regarding mannitol, it should be noted that there is a minimal dietary contribution (See Results). These findings are consistent with previous investigations, notably those by Camilleri et al. (21) estimating that over 70% of mannitol is absorbed before reaching the colon. Regarding the interpretation of our results, we observed a moderate correlation between the LMR calculated at the two intervals. This correlation has been observed in previous studies, such as that by Camilleri et al. (21) who noted an increase in LMRs in later urine collections, suggesting potentially higher permeability of the colon compared to the small intestine. They hypothesized that a significant portion of the mannitol may have been absorbed by the small intestine, reducing its availability for absorption in the colon, unlike lactulose, according to our data.

A review of 24 studies concluded that the mean LMR in healthy individuals was 1.4, which is consistent with our findings (1.04 for the 0–6-hours interval and 1.43 for the 2–15-hours interval) (22). Controversy surrounds the establishment of cutoff value for LMR indicating altered intestinal permeability. Some studies set the normal value at 2.5 (23), while others used 3 (24), although this may depend on various factors such as the test procedure. Therefore, in this study, no cutoff value was considered for data evaluation. Instead, an outlier analysis was conducted to identify any anomalous results compared to the rest of the group, given that only healthy volunteers were involved. Volunteer 15 was exhibited unusual values for both the LMR and u-GIP in both intervals, as shown in **Figure 3**. The likelihood of this occurrence is 0.008%. Detecting an outlier with elevated values for both parameters is an intriguing finding that supports the feasibility of using u-GIP as a potential measure of intestinal permeability. However, further research and analyses involving a significant number of participants (both healthy and diseased) are necessary to confirm this correlation and determine the clinical value of u-GIP in assessing intestinal permeability.

Correlations between LMR and u-GIP parameters at both time intervals were evaluated, along with % Lac and u-GIP. Our analysis detected a moderate correlation between the LMR and u-GIP parameters during the 2–15-hours interval (R^2 =^ 0.5225) when considering the presence of an outlier. However, upon excluding this outlier, the correlation weakened, suggesting an insignificant relationship between the two variables. This observation is consistent with the expected outcomes in the healthy control group, where variations in intestinal permeability parameters were anticipated to be minimal. Interestingly, Ordiz et al. (25) mentioned in their study involving the L:M test in 1669 rural Malawian children, that the strong direct correlation between percentage lactulose and percentage mannitol excretion does not support the use of mannitol as a normalizing factor for lactulose. They suggested that the use of percentage lactulose excretion alone provides more information about gut integrity than the LMR. Conversely, when outliers were removed from the analysis of %Lac vs u-GIP for the 2–15-hours interval, a moderate correlation (R^2 =^ 0.4159) emerged. This finding suggests a potential association between lactulose and u-GIP excretion rates within this timeframe, indicating underlying physiological mechanisms that warrant further investigation.

In this study, significant similarities have been identified between lactulose and u-GIP. Both compounds exhibit comparable temporal patterns in urinary excretion, including similar times to first quantifiable urine sample and duration of excretion. Furthermore, comparing excretion across the studied intervals (0–6-hours and 2–15-hours) reveals slower absorption rates for both u-GIP and lactulose. These findings support the theory that gluten peptides are predominantly absorbed via the paracellular pathway, akin to lactulose, due to their substantially larger molecular size compared to molecules predominantly absorbed via the transcellular route in the small intestine.

One notable weakness of the study lies in the dietary conditions post the 6-hour mark, where participants resumed consuming varied foods. This variability in dietary intake among participants introduces uncertainty regarding its potential impact on the excretion dynamics of the substances tested. It remains unclear whether these dietary variations could have altered the results due to minor dietary transgressions. This factor collectively highlights the need for careful consideration and further exploration in future research to mitigate such potential biases.

While these results are promising and demonstrate the feasibility of an intestinal permeability test based on u-GIP, further studies involving individuals exhibiting gastrointestinal conditions that may suggest altered permeability are necessary to compare and validate u-GIP as a marker. One potential concern regarding the use of gluten in assessing intestinal damage, particularly in the context of celiac disease, should not pose a problem during the diagnosis, as a gluten challenge is essential for reliable diagnosis. Given that occasional gluten ingestion is common and often asymptomatic (8, 26), it can also be considered a valuable specialized tool for monitoring the progression of intestinal damage during the gluten-free diet. This can be achieved using urinary GIP after a single gluten intake and for characterizing clinical symptoms (presence or type of symptoms). Given the disparities observed in the dynamics of this cohort comprising 15 volunteers, despite standardized conditions minimizing other dietary factors, exploring the potential for intraindividual variability justifies further investigation. Further clinical studies on individuals with gastrointestinal conditions where changes in permeability are potentially present will ascertain the utility of this approach in estimating intestinal permeability to food antigens.

## Data availability statement

The raw data supporting the conclusions of this article will be made available by the authors, without undue reservation.

## Ethics statement

The studies involving humans were approved by Ethics Committee of Virgen del Rocío and Virgen Macarena University Hospitals (n. 1308-N-23). The studies were conducted in accordance with the local legislation and institutional requirements. The participants provided their written informed consent to participate in this study.

## Author contributions

RR: Formal analysis, Funding acquisition, Investigation, Methodology, Validation, Visualization, Writing – original draft. MP: Formal analysis, Funding acquisition, Investigation, Methodology, Validation, Writing – review & editing. IM: Conceptualization, Formal analysis, Investigation, Methodology, Validation, Writing – review & editing. JL: Conceptualization, Formal analysis, Funding acquisition, Project administration, Supervision, Validation, Writing – review & editing. CS: Conceptualization, Funding acquisition, Project administration, Supervision, Writing – review & editing. ÁC: Conceptualization, Funding acquisition, Project administration, Resources, Supervision, Writing – review & editing.
